# Phenotypic spectrum and genetic analysis in the fatal cases of Schaaf-Yang syndrome: Two case reports and literature review

**DOI:** 10.1097/MD.0000000000020574

**Published:** 2020-07-17

**Authors:** Xuefei Chen, Xiaolu Ma, Chaochun Zou

**Affiliations:** a Department of Endocrinology; b Department of Neonatal Intensive Care Unit, The Children's Hospital, Zhejiang University School of Medicine, National Clinical Research Center for Child Health, Hangzhou, Zhejiang Province, China.

**Keywords:** congenital heart disease, *MAGEL2*, respiratory distress, Schaaf-Yang syndrome

## Abstract

**Rationale:**

Schaaf-Yang syndrome, a rare imprinted hereditary disease caused by *MAGEL2* variants, manifests as developmental delay/intellectual disability, neonatal hypotonia, feeding difficulties, contractures, and autism spectrum disorder.

**Patient concerns:**

Patient 1 and 2 were infant girls presenting facial dysmorphisms, contractures of interphalangeal joints, neonatal hypotonia, feeding difficulties, congenital heart diseases, and respiratory complications. Besides, Patient 2 presented with delayed psychomotor development.

**Diagnosis:**

Whole-exome sequencing was performed and heterozygous mutations of the *MAGEL2* gene were detected in the patients. They were diagnosed as Schaaf-Yang syndrome.

**Interventions:**

The patients received supportive treatment including mechanical ventilation, parenteral nutrition and gastric tube feeding.

**Outcomes:**

Whole-exome sequencing revealed de novo heterozygous c.1996dupC pathogenic mutations in the *MAGEL2* gene in the 2 patients. They died due to respiratory failure at the age of 20 days and 98 days, respectively.

**Lessons:**

Our results indicate that *MAGEL2* variants can cause congenital heart disease and fatal respiratory complications, broadening the phenotypic spectrum and adding to the fatal cases of Schaaf-Yang syndrome. We highly suggest that the *MAGEL2* gene should be added to gene-panels or gene-filters in next-generation sequencing-based diagnostics, which is of great significance for early diagnosis and early intervention of Schaaf-Yang syndrome patients.

## 1 Introduction

Schaaf-Yang syndrome (SYS) (OMIM 615547), also known as Prader-Willi-like syndrome, is a genetic disorder characterized by developmental delay and intellectual disability (DD/ID), neonatal hypotonia, feeding difficulties, contractures, and autism spectrum disorder (ASD). Mutations in the maternally imprinted, paternally expressed melanoma antigen L2 (*MAGEL2*) gene (OMIM 605283), located in the Prader-Willi critical region 15q11–15q13, are responsible for SYS. MAGEL2, a member of the MAGE family of ubiquitin ligase regulator, is a relatively GC-rich, single-exon gene that encodes a protein involved in endosomal protein recycling.^[1,2]^ It is exclusively expressed from the unmethylated paternal allele and only the mutation on the paternal allele leads to SYS.^[3]^ Unlike autosomal dominant inheritance, variants in the maternal imprinted allele can lead the phenotypes to skip several generations. However, for male individuals carrying a deleterious *MAGEL2* mutation, 50% of their offspring will be clinically affected.^[4]^

The most common phenotype of SYS is DD/ID that is present in almost 100% of the individuals for whom this information had been provided, followed by neonatal hypotonia and feeding difficulties (97%), then joint contractures (88%).^[5]^ Respiratory distress is another notable manifestation of SYS with a prevalence of 71%,^[5]^ while in the minority of cases, it can be particularly hazardous. Congenital heart disease accounts for a large proportion of the congenital defects in patients with Prader-Willi syndrome (PWS),^[6]^ which has a high cardiovascular risk and mortality.^[7]^ Nevertheless, to the best of our knowledge, congenital heart disease in patients with SYS has not been reported. It has been indicated that the phenotypic severity depended on the specific location of the variant, suggesting a genotype-phenotype correlation.^[5]^ Recently, Chitayat-Hall syndrome, a rare condition characterized by distal arthrogryposis, intellectual disability, dysmorphic features, hypopituitarism, and particularly growth hormone deficiency,^[8]^ was demonstrated to be another *MAGEL2*-related disorder.^[9,10]^ Compared with the relatively higher incidence of PWS cases, SYS cases especially the fatal cases of SYS were rarely reported. Herein, we reported 2 fatal cases of SYS with respiratory failure and congenital heart disease to expand the phenotypic spectrum, and reviewed the related literature to summarize the phenotypic and genetic characteristics in the fatal cases of SYS.

## 2 Methods

Two pediatric patients diagnosed in our unit from November 2017 to July 2019 were enrolled. The diagnosis was based on the phenotypes and genotypes with *MAGEL2* variants identified by whole-exome sequencing. After obtaining the informed consent from patients parents, genomic DNA was extracted from peripheral blood samples of the patients and their parents, and whole-exome sequencing was performed on Illumina HiSeq X ten platform. Detected *MAGEL2* mutations were subsequently confirmed by Sanger sequencing. For these patients, we investigated the history of birth, the developmental, physical, cognitive, neurological spectrum, and dysmorphic features through the collection of clinical data and communication with their parents. Height and weight standard deviation were evaluated according to the growth standardized values and curves of Chinese children.^[11]^ The clinical investigations and genetic analyses have been approved by the Medical Ethical Committee of the Children's Hospital of Zhejiang University School of Medicine.

### 2.1 Case 1

Patient 1 was the second child of healthy, nonconsanguineous parents, and her elder brother died due to leukemia at the age of 20 years. After an uneventful pregnancy, she was born at term by caesarean section due to intrauterine fetal distress. At birth, her weight was 3200 g (-1 SD to median), her length was 47 cm (-2 SD to -1 SD), and head circumference was 34.5 cm (median to +1 SD). The amniotic fluid was III degrees polluted. Apgar scores were 5 at 1 and 6 at 5 minutes. She was immediately treated with airway cleaning and artificially ventilated with the use of a bag-valve mask. However, she developed severe respiratory distress, prompting transfer to the neonatal intensive care unit. Seizures occurred shortly after birth, presenting as upper limb flexion and lower limb rigidity, and were controlled by midazolam. She was dependent on mechanical ventilation and parenteral nutrition in the first 15 days after birth due to respiratory distress and feeding difficulties.

**Figure 1:**
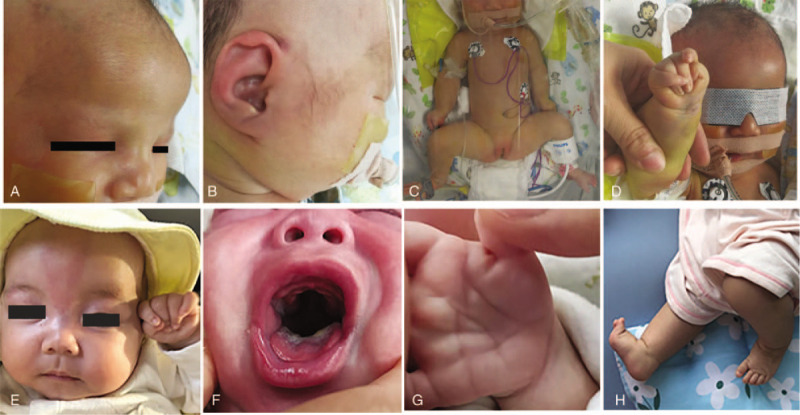
Clinical manifestations of the 2 patients with Schaaf-Yang syndrome. Patient 1: (A) Frontal bossing with a ridge over the metopic suture, hypoplastic supraorbital ridges and a prominent nasal root; (B) Large and abnormally shaped ears without auricles; (C) Short neck, short limbs, long and narrow thorax; (D) Adducted thumb and tapering fingers with the second and fifth fingers overlapping the third and fourth. Patient 2: (E) High forehead, full cheeks, a broad nasal root, a long philtrum and the micrognathia, adducted thumb and tapering digits with the second and fifth fingers overlapping the third and fourth; (F) A large mouth, thickened gums and a high arched palate; (G) Small hands with abnormal palmar creases; (H) Small club feet.

**Figure 2:**
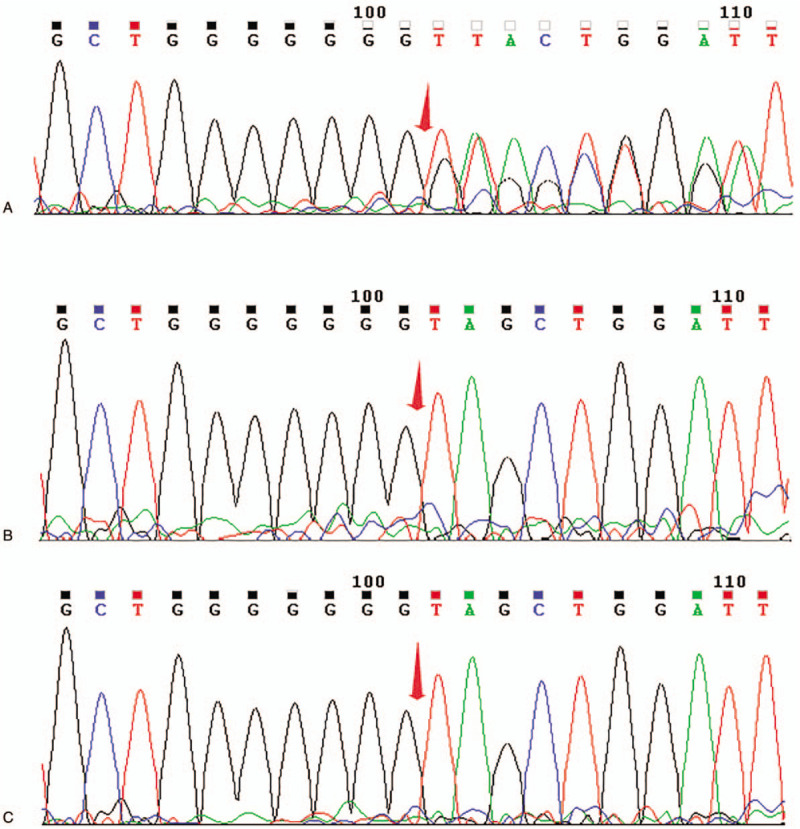
Sanger sequencing chromatograms of *MAGEL2* mutations in the family of case 1. (A) Heterozygous c.1996dupC mutation in *MAGEL2* in patient 1; (B) No mutations detected in her father; (C) No mutations detected in her mother.

The patient had facial dysmorphisms including a high forehead with a ridge over the metopic suture, hypoplastic supraorbital ridges, a prominent nasal root, large and abnormally shaped ears without auricles. Furthermore, she manifested short neck, long and narrow thorax, short limbs, joint contractures in the form of camptodactyly with the second and fifth fingers overlapping the third and fourth, adducted thumbs and tapering fingers (Fig. 1A-D). Neurological examination revealed hypotonia with disappeared foraging reflex, sucking reflex, hugging reflex, holding reflex and weakened swallowing reflex. Echocardiography showed the ventricular septal defect with a diameter of 4.0 mm, atrial septal defect of 2.9 mm and pulmonary hypertension. Other examinations including brain magnetic resonance imaging, long bone X-ray of the limbs, ultrasonography of the liver, bile, pancreas, spleen and urinary system, thyroid, liver and kidney function yielded normal results. Whole-exome sequencing identified a de novo heterozygous c.1996dupC variant in exon 1 of the *MAGEL2* gene in the patient, which was confirmed by Sanger sequencing (Fig. 2). Biological information analysis implied frameshift and premature termination in the 666th amino acid after this duplication (p.Q666Pfs∗47), and it was regarded as a pathogenic variant. The patient showed progressive poor response and respiratory difficulties in the intensive care unit, and her parents decided to abandon the treatment in consideration of poor prognosis. She died due to respiratory failure at the age of 20 days.

### 2.2 Case 2

Patient 2 was born by vaginal delivery at term with the weight of 3400 g (median to +1 SD) and length of 51 cm (median to +1 SD). Her parents were healthy and unrelated, and family history was unremarkable. Apgar score was 9 at 1 and 7 at 5 minutes. The amniotic fluid was II degrees polluted and meconium inhaled was found in the neonate. Although airway cleaning and artificial ventilation with the use of a bag-valve mask were performed on the patient, she manifested as the poor response, weak crying, cyanosis, hypotonic lower limbs and hypertonic upper limbs. The newborn was transferred to the neonatal intensive care unit for neonatal asphyxia and neurological symptoms, and was dependent on assisted ventilation in the first 3 days. She had poor sucking and oropharyngeal dysphagia and required gastric tube feeding during the first 27 days.

**Figure 3:**
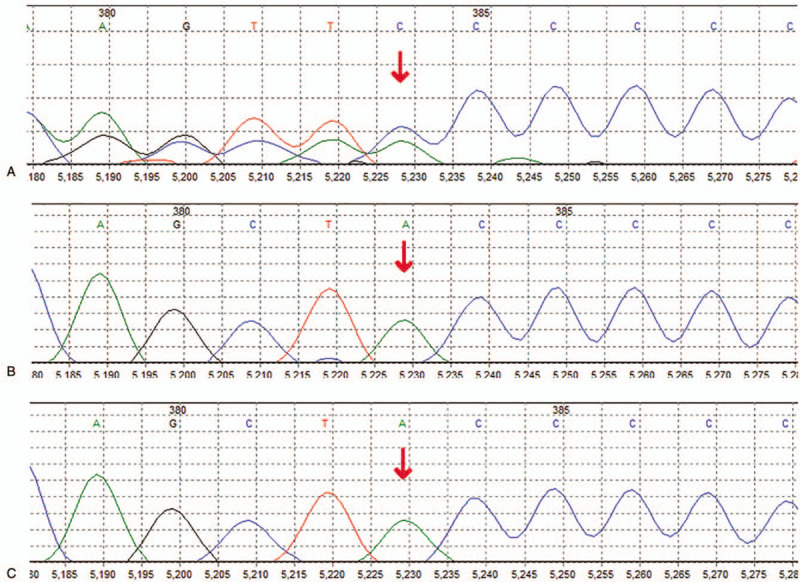
Sanger sequencing chromatograms of *MAGEL2* mutations in the family of case 2. (A) Heterozygous c.1996dupC mutation in *MAGEL2* in patient 2; (B) No mutations detected in her father; (C) No mutations detected in her mother.

Clinical examination disclosed the presence of high forehead, full cheeks, almond-shaped eyes, a broad nasal root, a long philtrum, a large mouth, thickened gums, a high arched palate and micrognathia, tapering digits and interphalangeal joint contractures similar to patient 1, small hands with abnormal palmar creases, and small club feet (Fig. 1E-H). At the age of 2 and a half months old, the patient manifested hypertonia of upper limbs and high muscle tension of shoulder and back. She could not lie flat and often leaned to one side. She had poor head control and could not elevate her head for a long time. She exhibited poor eye tracking and sometimes smiled unconsciously without eye contact with others. Laryngomalacia and symptoms of gastroesophageal reflux were noted in this patient. Echocardiography showed the ventricular septal defect of 3.2 mm and patent foramen ovale. Other examinations including brain magnetic resonance imaging, genetic metabolic screening, chromosomal microarray analysis and DNA methylation analysis within the Prader-Willi critical region revealed no abnormity. A de novo heterozygous c.1996dupC (p.Gln666fs) pathogenic variant in the *MAGEL2* gene was detected in the proband, and was confirmed by Sanger sequencing (Fig. 3). At the age of 78 days, the patient presented with severe aspiration pneumonia due to gastroesophageal reflux. She developed respiratory failure and unexplained cerebral edema rapidly, and was admitted to the intensive care unit. Despite persistent mechanical ventilation and mild hypothermia therapy, the patient died at age 98 days.

## 3 Discussion

Schaaf et al first described the phenotypes of patients with truncating mutations in *MAGEL2* on the paternal allele.^[3]^*MAGEL2* has 51% amino acid sequences similarity to necdin, a protein implicated in the terminal differentiation of neurons and essential for axonal outgrowth.^[12]^*MAGEL2* is mainly expressed in the central nervous system, especially in the hypothalamus,^[13,14]^ important for neuronal development.^[2,15]^ It also plays a key role in modulating bone remodeling and mass, and is required for normal strength, endurance, and the maintenance of muscle.^[16,17]^ Variants in *MAGEL2* can cause central nervous system retardation and dysmorphic features.^[18]^ Both our patients presented with variable degrees of neurodevelopmental delay including seizures and weakened primitive reflexes in patient 1 and psychomotor delay in patient 2, together with contractures and neonatal hypotonia, consistent with the phenotypes caused by *MAGEL2* variants.

Our patients carried the mutational hotspot c.1996dupC mutations and bore good phenotypic resemblance with SYS. They presented with characteristic dysmorphia, neonatal hypotonia, respiratory distress, feeding difficulties, particularly joint contractures, which were observed among the majority of patients with *MAGEL2* variants.^[5,10]^ ASD, common in *MAGEL2-*mutated patients, has not been evaluated due to the limitation of age. While SYS and PWS share an appreciable amount of phenotypes including DD/ID, hypotonia, feeding difficulties and hypogonadism,^[19]^ there exist some phenotypes of SYS distinct from those of PWS. Patients with SYS manifest a much higher prevalence of joint contractures (88%) and ASD (78%),^[20]^ but a lower prevalence of hyperphagia and morbid obesity than that seen in PWS patients.^[5,21]^ It was recently reported that compared to individuals with PWS, patients with SYS displayed a more profound level of intellectual disability and a more severe form of developmental delay.^[5,22]^ Though both our patients manifested interphalangeal joint contractures which helped the diagnosis of SYS, it is less rigorous to distinguish SYS from PWS only based on the phenotypes, as no specific feature appears to be exclusive of one or the other condition. Whole-exome sequencing has been further performed and truncating variants in *MAGEL2* were detected in the patients. According to the typical features and molecular genetic test results, the diagnosis of SYS was identified. Hence, we highlight that *MAGEL2* sequencing should be considered in the context of PWS-like phenotypes after excluding PWS by methylation PCR to assist the diagnosis of SYS.

Worthy of note is the phenotype of congenital heart disease present in our patients but not described in other patients with *MAGEL2* variants. The 2 patients shared the common congenital heart disease of the ventricular septal defect. *MAGEL2* was described to be paternally expressed in heart of pigs,^[23]^ but the mechanism of the *MAGEL2* variants to the congenital heart disease, for example, whether *MAGEL2* variants perturb the normal program of cardiac development, remains unclear. Moreover, congenital heart disease was shown to have associations with neurodevelopmental abnormalities such as intellectual disability, language deficits, ASD, and motor skills deficits,^[24]^ but the underlying causes remain poorly defined. It is uncertain whether there exists a correlation of the neurodevelopmental abnormalities with the congenital heart disease in our patients, and further study is needed.

Through the literature review, we found 13 fatal cases of SYS reported previously,^[4,15,25–27]^ and we included a simplified summary of the phenotypic and genetic characteristics in these cases in Table 1. We discovered that all the decedents manifested with contractures. Other phenotypes including feeding difficulties, DD/ID and neonatal hypotonia were also notable, accounting for 100%, 100%, and 90% in the patients evaluated, respectively. Our results indicate that severe arthrogryposis multiplex congenita and respiratory failure are the main cause of early death in the SYS patients. All the 5 individuals with c.1996delC mutation died prenatally or soon after birth due to severe arthrogryposis multiplex congenita and fetal akinesia, suggesting that c.1996delC mutation is likely to be fatal and is responsible for severe arthrogryposis, which is consistent with the previous findings.^[15]^ Another important variant associated with fatal cases of SYS is c.1996dupC mutation which was detected in 7 patients of the cohort, recapitulating that c.1996dupC mutation displays a more severe phenotype than other truncating mutations (except for c.1996delC mutation).^[5]^

**Table 1:**
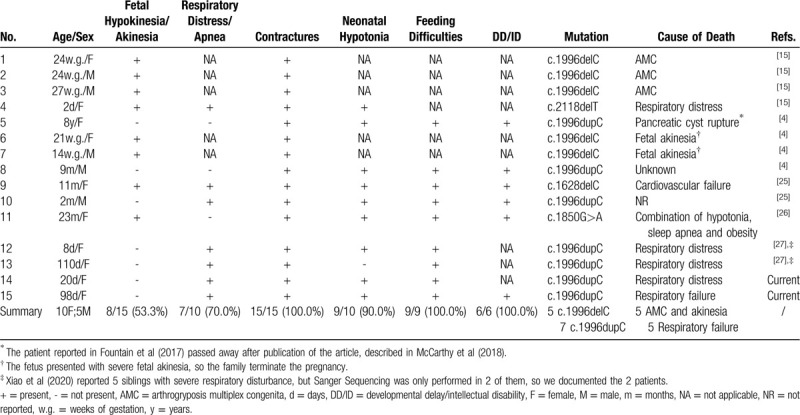
Clinical features and *MAGEL2* variants in fatal cases of Schaaf-Yang syndrome.

There were 5 deaths due to respiratory complications, and 4 of them carried c.1996dupC mutations, suggesting that patients with c.1996dupC variants have a higher prevalence of respiratory dysfunction compared to those with other variants. Respiratory distress is a common manifestation of SYS, as 55% of SYS patients were described to require mechanical ventilation in the first 2 months of life.^[5]^ The underlying mechanism of respiratory complications of SYS could be multiple. Firstly, while predominantly expressed in the brain, *MAGEL2* is also expressed in the fetal lung,^[18]^ and moderately expressed in abdominal wall muscle and diaphragm in the mouse model,^[16]^ indicating that loss of function may lead to respiratory difficulties. Hypotonia in the diaphragm and abdominal wall muscle is another cause of respiratory disease, as it can result in weakened cough reflex, increasing the risk of aspiration pneumonia. This may be the cause of respiratory failure in patient 2, who was further influenced by gastroesophageal reflux. Furthermore, as *MAGEL2* is primarily expressed in the hypothalamus, respiratory complications are also presumed to be secondary to CNS dysfunction. Although the majority of SYS patients can survive with the disease, fatal cases cannot be ignored. Respiratory issues could be extremely hazardous to cause death in early childhood of SYS patients.

In conclusion, the phenotypes of SYS continue to evolve, and further investigations of individuals with variants in *MAGEL2* are necessary. The congenital heart disease identified in our patients has not been reported before, broadening the phenotypic spectrum of SYS. We also emphasize that the respiratory complications could be fatal and should be monitored carefully. We suggest that *MAGEL2* gene should be added to gene-panels or gene-filters in next-generation sequencing-based diagnostics, which is of great significance for early diagnosis of patients with SYS.

## Acknowledgments

We are indebted to the patients and their families for allowing us to use the data.

## Author contributions

Xuefei Chen wrote the original manuscript. Xiaolu Ma performed the data collection. Chaochun Zou reviewed and revised the manuscript. All authors read and approved the manuscript.

## References

[1] Tacer KF Potts PR Cellular and disease functions of the Prader-Willi Syndrome gene MAGEL2. Biochem J 2017;474:2177–90. 28626083 10.1042/BCJ20160616 PMC5594744

[2] Hao YH Fountain MD Jr Fon Tacer K USP7 acts as a molecular rheostat to promote WASH-dependent endosomal protein recycling and is mutated in a human neurodevelopmental disorder. Mol Cell 2015;59:956–69. 26365382 10.1016/j.molcel.2015.07.033 PMC4575888

[3] Schaaf CP Gonzalez-Garay ML Xia F Truncating mutations of MAGEL2 cause Prader-Willi phenotypes and autism. Nat Genet 2013;45:1405–8. 24076603 10.1038/ng.2776 PMC3819162

[4] Fountain MD Aten E Cho MT The phenotypic spectrum of Schaaf-Yang syndrome: 18 new affected individuals from 14 families. Genet Med 2017;19:45–52. 27195816 10.1038/gim.2016.53 PMC5116288

[5] McCarthy J Lupo PJ Kovar E Schaaf-Yang syndrome overview: report of 78 individuals. Am J Med Genet A 2018;176:2564–74. 30302899 10.1002/ajmg.a.40650 PMC6585857

[6] Torrado M Foncuberta ME Perez MF Change in prevalence of congenital defects in children with Prader-Willi syndrome. Pediatrics 2013;131:e544–9. 23296430 10.1542/peds.2012-1103

[7] Einfeld SL Kavanagh SJ Smith A Mortality in Prader-Willi syndrome. Am J Ment Retard 2006;111:193–8. 16597186 10.1352/0895-8017(2006)111[193:MIPS]2.0.CO;2 PMC2422866

[8] Chitayat D Hall JG Couch RM Syndrome of mental retardation, facial anomalies, hypopituitarism, and distal arthrogryposis in sibs. Am J Med Genet 1990;37:65–70. 2240046 10.1002/ajmg.1320370116

[9] Jobling R Stavropoulos DJ Marshall CR Chitayat-Hall and Schaaf-Yang syndromes:a common aetiology: expanding the phenotype of MAGEL2-related disorders. J Med Genet 2018;55:316–21. 29599419 10.1136/jmedgenet-2017-105222

[10] Patak J Gilfert J Byler M MAGEL2-related disorders: a study and case series. Clin Genet 2019;96:493–505. 31397880 10.1111/cge.13620 PMC6864226

[11] Li H Growth standardized values and curves based on weight, length/height and head circumference for Chinese children under 7 years of age. Zhonghua Er Ke Za Zhi 2009;47:173–8. 19573429

[12] Lee S Walker CL Karten B Essential role for the Prader-Willi syndrome protein necdin in axonal outgrowth. Hum Mol Genet 2005;14:627–37. 15649943 10.1093/hmg/ddi059

[13] Kozlov SV Bogenpohl JW Howell MP The imprinted gene Magel2 regulates normal circadian output. Nat Genet 2007;39:1266–72. 17893678 10.1038/ng2114

[14] Maillard J Park S Croizier S Loss of Magel2 impairs the development of hypothalamic Anorexigenic circuits. Hum Mol Genet 2016;25:3208–15. 27288456 10.1093/hmg/ddw169 PMC5179922

[15] Mejlachowicz D Nolent F Maluenda J Truncating mutations of MAGEL2, a gene within the Prader-Willi locus, are responsible for severe arthrogryposis. Am J Hum Genet 2015;97:616–20. 26365340 10.1016/j.ajhg.2015.08.010 PMC4596890

[16] Kamaludin AA Smolarchuk C Bischof JM Muscle dysfunction caused by loss of Magel2 in a mouse model of Prader-Willi and Schaaf-Yang syndromes. Hum Mol Genet 2016;25:3798–809. 27436578 10.1093/hmg/ddw225

[17] Baraghithy S Smoum R Drori A Magel2 modulates bone remodeling and mass in prader-willi syndrome by affecting oleoyl serine levels and activity. J Bone Miner Res 2019;34:93–105. 30347474 10.1002/jbmr.3591

[18] Lee S Kozlov S Hernandez L Expression and imprinting of MAGEL2 suggest a role in Prader-willi syndrome and the homologous murine imprinting phenotype. Hum Mol Genet 2000;9:1813–9. 10915770 10.1093/hmg/9.12.1813

[19] Fountain MD Schaaf CP Prader-Willi syndrome and Schaaf-Yang syndrome: neurodevelopmental diseases intersecting at the MAGEL2 Gene. Diseases 2016;4:2. 10.3390/diseases4010002 PMC5456300 28933382

[20] Bennett JA Germani T Haqq AM Autism spectrum disorder in Prader-Willi syndrome: a systematic review. Am J Med Genet A 2015;167a:2936–44. 26331980 10.1002/ajmg.a.37286

[21] Cassidy SB Schwartz S Miller JL Prader-Willi syndrome. Genet Med 2012;14:10–26. 22237428 10.1038/gim.0b013e31822bead0

[22] Thomason MM McCarthy J Goin-Kochel RP Neurocognitive and neurobehavioral phenotype of youth with Schaaf-Yang syndrome. J Autism Dev Disord 2018. 10.1007/s10803-018-3775-7 30343463

[23] Guo L Qiao M Wang C Imprinting analysis of porcine MAGEL2 gene in two fetal stages and association analysis with carcass traits. Mol Biol Rep 2012;39:147–55. 21633897 10.1007/s11033-011-0719-0

[24] Zaidi S Brueckner M Genetics and genomics of congenital heart disease. Circ Res 2017;120:923–40. 28302740 10.1161/CIRCRESAHA.116.309140 PMC5557504

[25] Tong W Wang Y Lu Y Whole-exome sequencing helps the diagnosis and treatment in children with neurodevelopmental delay accompanied unexplained dyspnea. Sci Rep 2018;8:5214. 29581464 10.1038/s41598-018-23503-2 PMC5980106

[26] Kleinendorst L Pi Castan G Caro-Llopis A The role of obesity in the fatal outcome of Schaaf-Yang syndrome: early onset morbid obesity in a patient with a MAGEL2 mutation. Am J Med Genet A 2018;176:2456–9. 30238631 10.1002/ajmg.a.40486

[27] Xiao B Ji X Wei W A recurrent variant in MAGEL2 in five siblings with severe respiratory disturbance after birth. Mol Syndromol 2020;10:286–90. 32021601 10.1159/000501376 PMC6997796

